# Systemic corticosteroids for community-acquired pneumonia in adults

**DOI:** 10.1186/cc10646

**Published:** 2012-03-20

**Authors:** RJ Pugh, N Roy

**Affiliations:** 1 Glan Clwyd Hospital, Rhyl, UK

## Introduction

We aimed to evaluate evidence from randomised controlled trials (RCTs) investigating the effect of systemic corticosteroids in adults with community-acquired pneumonia (CAP). Observational data suggest that corticosteroids may decrease mortality in severe CAP [1], and several large RCTs have been published since the recent Cochrane review [2].

## Methods

A systematic review of the literature: Cochrane Central Register for Controlled Clinical Trials, MEDLINE, EMBASE and SCOPUS, and reference lists of original studies and reviews. Data were collated and analysed using Review Manager v5.1.

## Results

A total of 254 RCTs were identified. Seven met inclusion criteria, totalling 806 patients. Studies varied in methodology, participants, interventions, and outcome measures. Where meta-analysis was possible, data are presented in Table 1 (outcomes: hospital mortality, 30-day mortality, hospital length of stay, superinfection, hyperglycaemia). Excepting hyperglycaemia, effect estimates were not statistically significant. Two small studies (*n *= 46 and *n *= 30) concentrated on severe CAP (using ATS and BTS criteria); one study found a statistically significant reduction in mortality, lengths of stay and duration of mechanical ventilation in the steroid group, but similar improvements in the other study, and in a large subgroup of patients with severe CAP in another study (*n *= 93) were not found. Significant reductions in inflammatory markers in the week following initiation of steroid treatment were found in six studies.

**Table 1:** Meta-analysis of clinical outcomes

Outcome	Number of studies	Population	Effect
Hospital mortality	5	537	OR 0.65
30-day mortality	3	562	OR 0.90
Hospital LOS	2	244	MD -1.52
Superinfection	3	563	OR 1.24
Hyperglycaemia	2	517	OR 2.69*

LOS, length of stay. *P < 0.0001.

## Conclusion

Systemic corticosteroid administration as adjunctive treatment for CAP does not appear to improve relevant clinical outcomes, regardless of severity, and is associated with significantly increased incidence of hyperglycaemia.

## References

[B1] Garcia-Vidal Eur Respir J 2007 30 951 956 10.1183/09031936.00027607 17690125

[B2] Chen Cochrane Database of Systematic Reviews 2011 3

